# Giant Viruses of Amoebas: An Update

**DOI:** 10.3389/fmicb.2016.00349

**Published:** 2016-03-22

**Authors:** Sarah Aherfi, Philippe Colson, Bernard La Scola, Didier Raoult

**Affiliations:** ^1^Unité de Recherche sur les Maladies Infectieuses et Tropicales Emergentes, UM63 Centre National de la Recherche Scientifique 7278 Institut de Recherche pour le Développement 198 Institut National de la Santé et de la Recherche Médicale U1095, Aix-Marseille UniversitéMarseille, France; ^2^Institut Hospitalo-Universitaire Méditerranée Infection, Assistance Publique-Hôpitaux de Marseille, Centre Hospitalo-Universitaire Timone, Pôle des Maladies Infectieuses et Tropicales Clinique et Biologique, Fédération de Bactériologie-Hygiène-VirologieMarseille, France

**Keywords:** giant virus, mimivirus, amoeba, *Acanthamoeba*, *Megavirales*, virophage, 4th TRUC

## Abstract

During the 12 past years, five new or putative virus families encompassing several members, namely *Mimiviridae, Marseilleviridae*, pandoraviruses, faustoviruses, and virophages were described. In addition, *Pithovirus sibericum* and *Mollivirus sibericum* represent type strains of putative new giant virus families. All these viruses were isolated using amoebal coculture methods. These giant viruses were linked by phylogenomic analyses to other large DNA viruses. They were then proposed to be classified in a new viral order, the *Megavirales*, on the basis of their common origin, as shown by a set of ancestral genes encoding key viral functions, a common virion architecture, and shared major biological features including replication inside cytoplasmic factories. *Megavirales* is increasingly demonstrated to stand in the tree of life aside *Bacteria, Archaea*, and *Eukarya*, and the megavirus ancestor is suspected to be as ancient as cellular ancestors. In addition, giant amoebal viruses are visible under a light microscope and display many phenotypic and genomic features not found in other viruses, while they share other characteristics with parasitic microbes. Moreover, these organisms appear to be common inhabitants of our biosphere, and mimiviruses and marseilleviruses were isolated from human samples and associated to diseases. In the present review, we describe the main features and recent findings on these giant amoebal viruses and virophages.

## Introduction

Viruses were first described at the end of the nineteenth century as ultrafilterable and submicroscopic infectious agents (Beijerinck, 1898; Loeffler and Frosch, 1898). At that time, they were considered to be entities smaller than microbes, and they were mainly defined on the basis of negative criteria, including the absence of both DNA or RNA or components from the translation apparatus (Lwoff, 1957; Lwoff and Tournier, 1966). The discovery of Mimivirus in 2003 challenged this paradigm and fostered new debates on the definition and classification of viruses (Raoult et al., 2004; Raoult and Forterre, 2008; Raoult, 2014). In fact, Mimivirus was visible under a light microscope and its gene content was dramatically broader than that of other viruses, with as many genes as small bacteria. Moreover, some of these genes suggest a relative autonomy from their host cell for transcription and even translation (Raoult et al., 2004). Mimivirus was serendipitously isolated using coculture on *Acanthamoeba polyphaga*, a culture strategy that consists in inoculating samples onto an axenic amoebal culture and was implemented to grow microbes (Rowbotham, 1983). Thus, Mimivirus was discovered by bacteriologists and not by virologists (La Scola et al., 2003).

Subsequently, over the past 12 years, giant viruses were hunted using amoebal co-culture methods. As of 2015, five new or putative families of viruses infecting amoebas were described, which encompass members from giant viral families *Mimiviridae* and *Marseilleviridae*, pandoraviruses, faustoviruses, and mimivirus virophages, and *Pithovirus sibericum* and *Mollivirus sibericum* represent type strains of putative new giant virus families (La Scola et al., 2008; Desnues and Raoult, 2012; Colson et al., 2013a,d; Philippe et al., 2013; Legendre et al., 2014, 2015; Reteno et al., 2015). Giant amoebal viruses were linked by phylogenomic analyses to nucleocytoplasmic large DNA viruses (NCLDV), a group of double-stranded (ds) DNA viruses coined in 2001 that includes poxviruses, ascoviruses, iridoviruses, asfarviruses, and phycodnaviruses (Iyer et al., 2001; Yutin et al., 2009). Amoebal giant viruses and these dsDNA viruses were shown to share a small set of nine core genes, including five found in all of their genomes, as well as a larger subset of almost 200 genes shared by at least two NCLDV families. Moreover, these viruses were shown to share a common ancestor whose genome was inferred to harbor about 50 conserved genes, and they were suspected to have an early origin, concomitant with eukaryogenesis (Yutin et al., 2009; Yutin and Koonin, 2012). NCLDV families were proposed in 2013 to be reclassified in a new viral order, the *Megavirales*, on the basis of their common origin as shown by a large set of ancestral genes encoding key viral functions, a common virion architecture, and shared major biological features including replication inside cytoplasmic factories (Colson et al., 2013a). The order *Megavirales* encompasses viruses with a jelly roll capsid whose diameter is >150 nm and a genome larger than 100 kb that encodes all five former NCLDV class I genes (namely, major capsid protein, D5 helicase, DNA polymerase B, A32-like packaging ATPase, and Very Late Transcription Factor 3). However, in pandoraviruses and *P. sibericum* that were discovered since 2013, virion architecture differs and jelly roll capsid was not detected (Philippe et al., 2013; Klose and Rossmann, 2014; Legendre et al., 2014). In the present review, the main features of amoebal giant viruses and virophages are described.

## Mimiviruses and virophages

**Figure 1:**
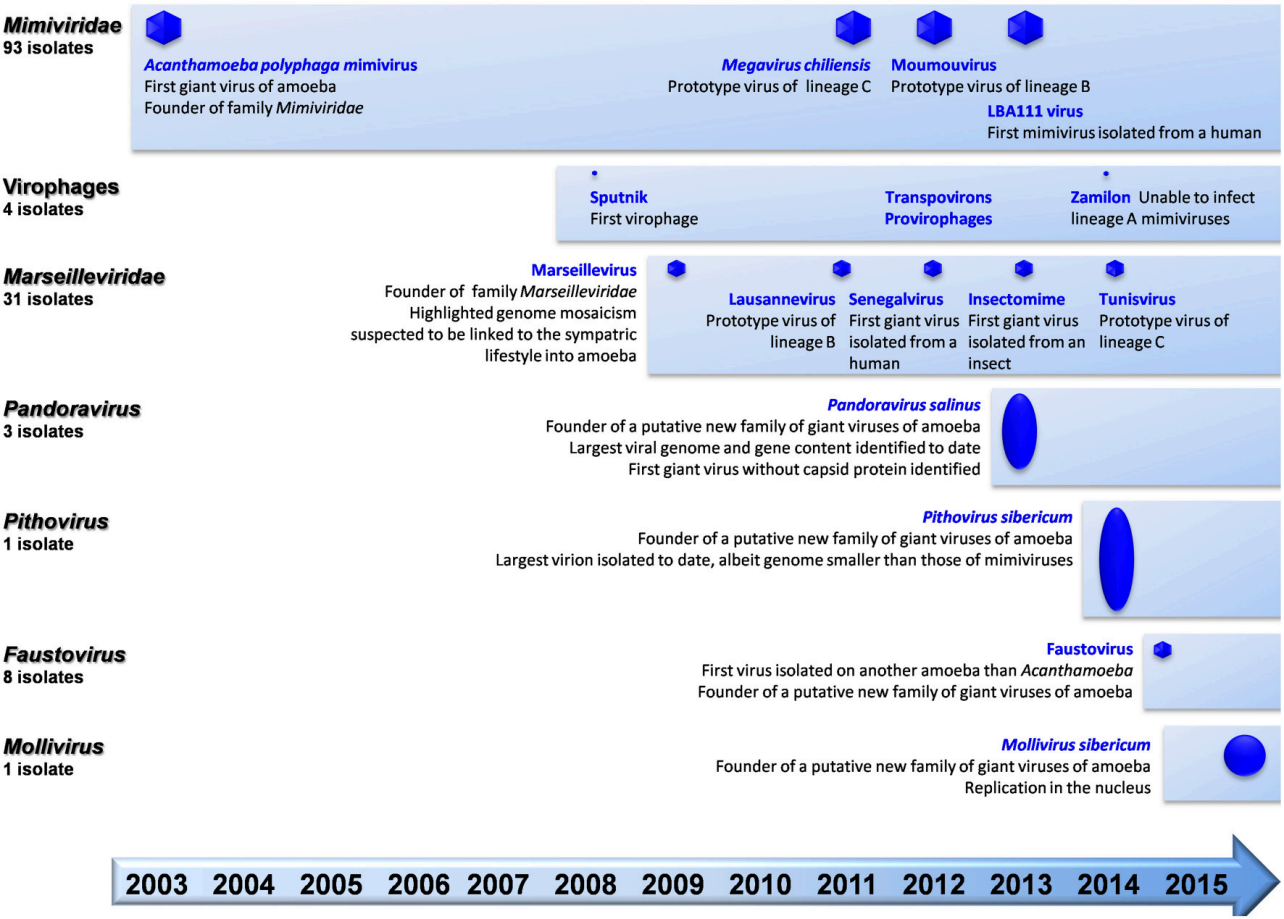
**Schematic of the chronological order of discovery of major representatives of giant viruses of amoeba and virophages, and current number of isolates per family**.

**Figure 2:**
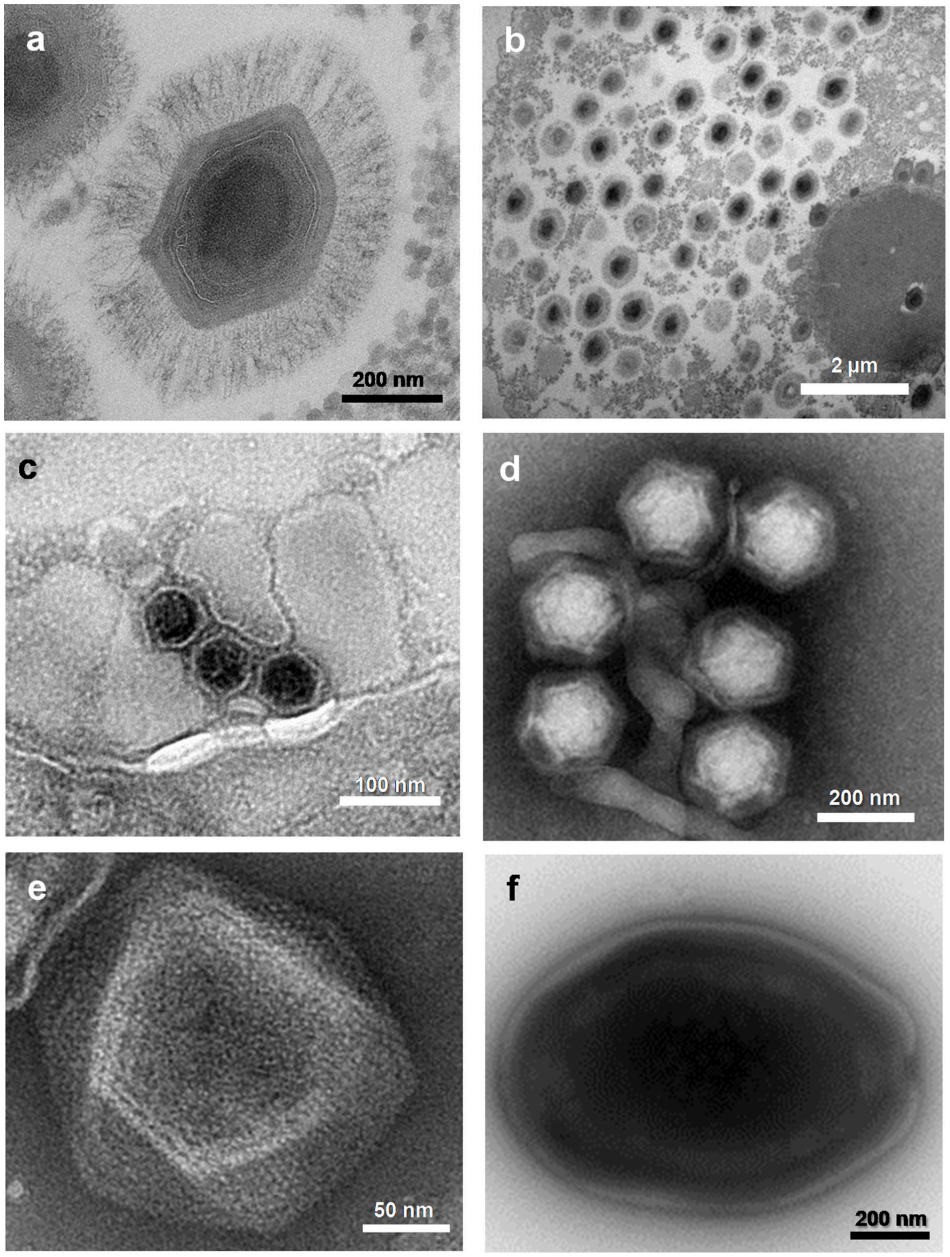
**Electron micrographs of viruses belonging to five families or putative families of viruses of amoebas**. **(A)** Mimivirus; **(B)** Mimivirus viral factory in *Acanthamoeba polyphaga*; **(C)** Marseillevirus; **(D)** Virophages; **(E)** Faustovirus; **(F)** *Pandoravirus* sp. These viruses were isolated at URMITE, IHU Méditerranée Infection.

**Figure 3:**
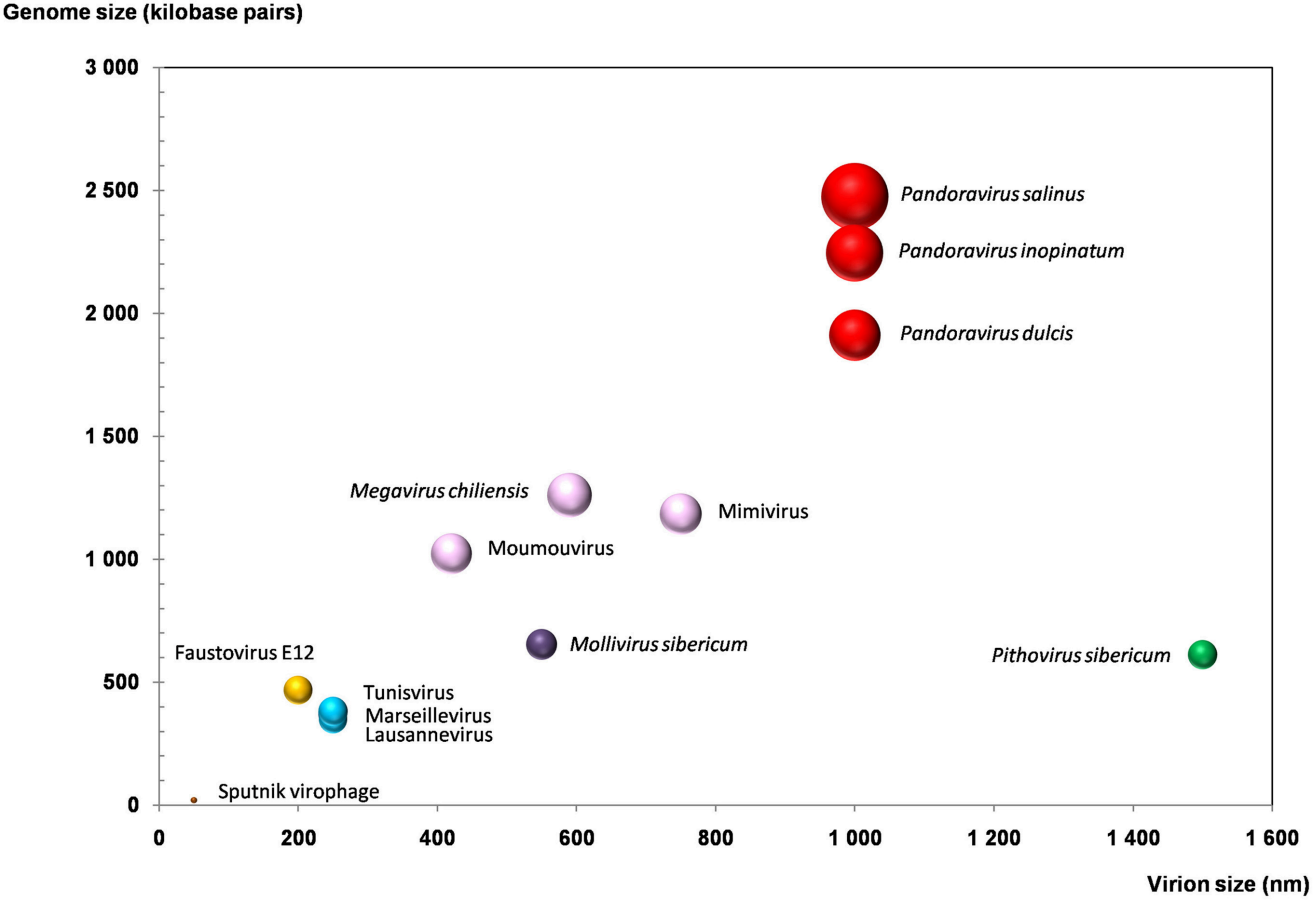
**Plot of the virion and genome sizes from representatives of giant viruses and virophages of amoebas**. Bubble size is proportional to the number of genes in the viral genome.

**Figure 4:**
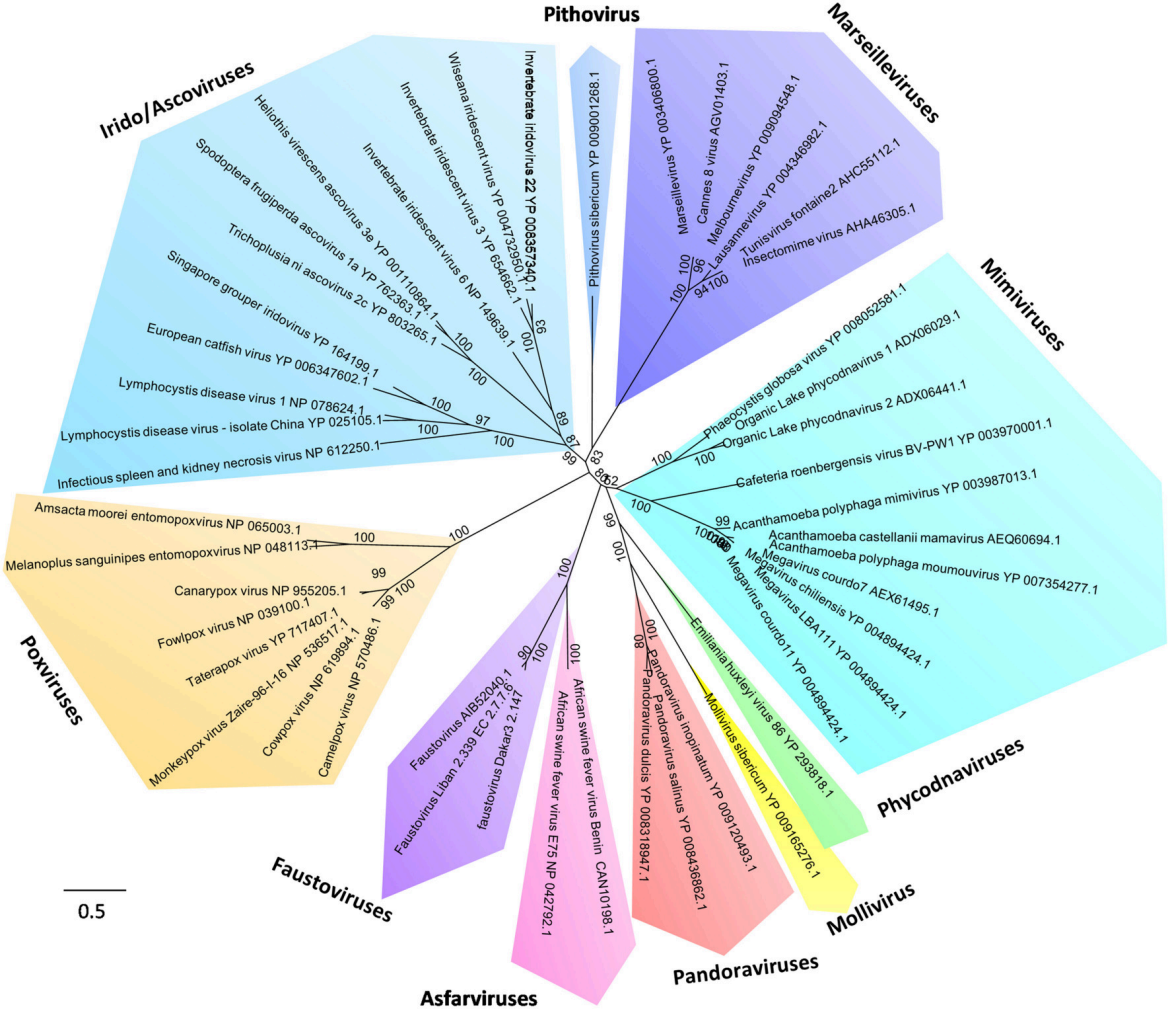
**Phylogeny reconstruction based on the RNA-dependent RNA polymerase from ***Megavirales*** representatives**. The sequences were aligned with the Muscle software (Edgar, 2004); the tree was built using the Maximum Likelihood method with the FastTree program (Price et al., 2010).

**Figure 5:**
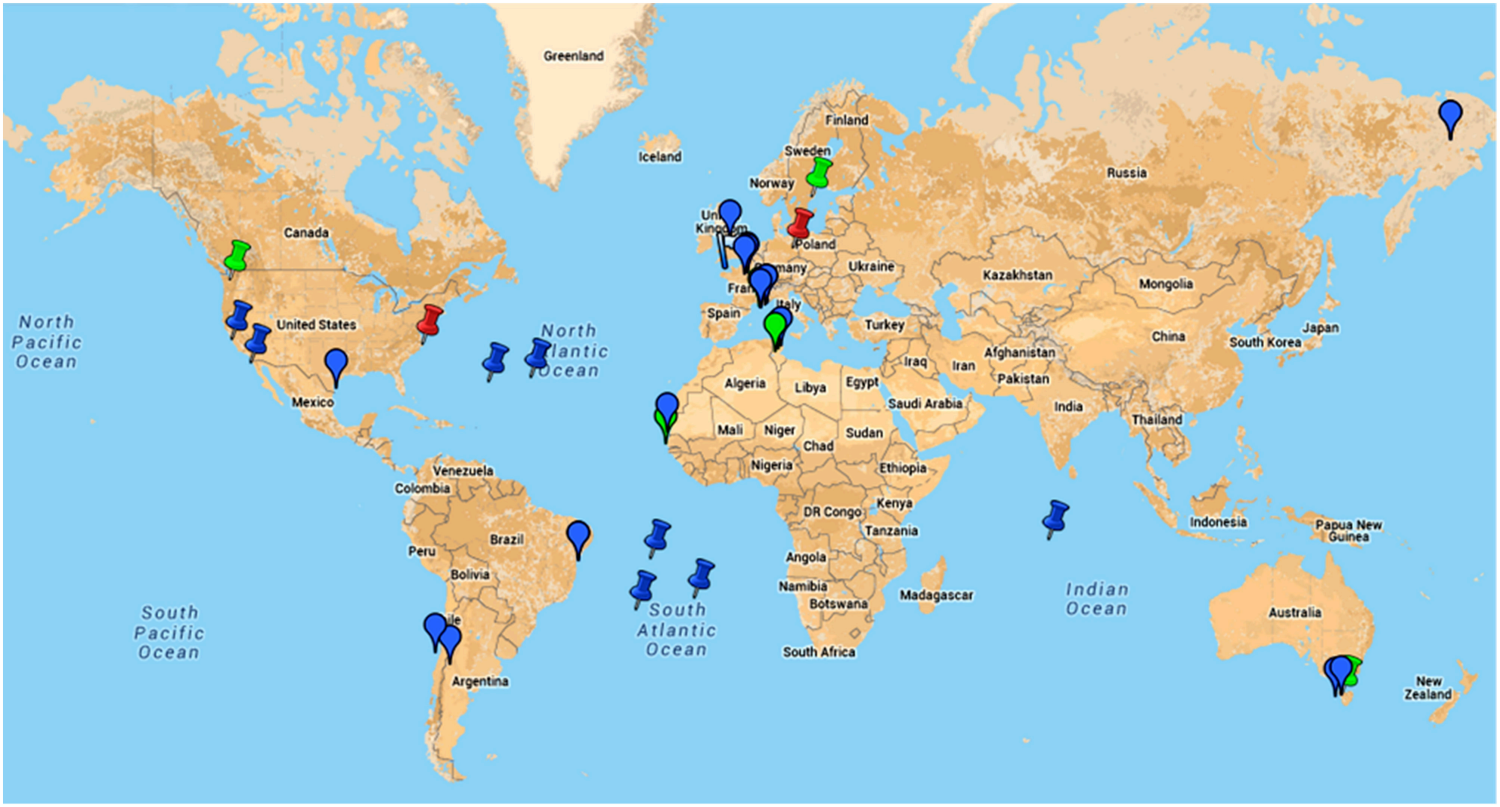
**Google map of locations of samples from where giant viruses of amoebas were isolated or in which metagenomic reads related to these viruses were detected**. 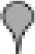, indicates location of samples from which an amoebal virus was isolated; 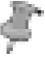, indicates location of samples from which reads related to an amoebal virus were generated by metagenomics; 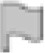, indicates the discovery of giant viral particles for which a virus could not be isolated. Blue color indicates environmental samples; green color indicates human samples; red color indicates animal (non-human) samples. This figure is a screenshot of a goggle map that is freely available at the following URL: https://www.google.com/maps/d/edit?mid=zA3X4ljlz-uM.kFSrbnCtoBLc.

*A. polyphaga* mimivirus (APMV), the pioneer representative of giant viruses of amoebas, was isolated in 1992 from a water sample collected in England in an air-conditioning system, while investigating a pneumonia outbreak (Table 1; Figures 1–5; La Scola et al., 2003). APMV isolation was performed by co-culturing on *A. polyphaga*, a strategy implemented primarily to retrieve “*Legionella*-like pathogens.” Mimivirus virions were first mistaken as bacteria, as they could be visualized by optical microscopy and resembled Gram-positive cocci. They were only identified as viral particles in 2003 by visualizing their icosahedral capsid by electron microscopy, then named Mimivirus to stress that they were viruses mimicking microbes (La Scola et al., 2003; Raoult et al., 2004, 2007). APMV founded the family *Mimiviridae*. Mimivirus virions were shown to have 500 nm-large capsids and 75 nm-long fibrils. These fibers have a unique structure amongst viruses (Klose et al., 2015) and were recently shown to allow attachment via glycans on different organisms including bacteria, arthropods and fungi (Rodrigues et al., 2015). In addition, genome sequencing revealed a dsDNA harboring 1.2 mega base pairs (Mbp) and 979 putative genes (Raoult et al., 2004; Legendre et al., 2010). Some of these genes had never been previously identified in viruses, like those encoding proteins involved in translation, including amino-acyl tRNA synthetases, and translation factors. Some amino-acyl tRNA synthetases were found to be functional (Abergel et al., 2007). In addition, the expression of Mimivirus genes related to translation was found to vary according to nutrient availability (Silva et al., 2015). Other Mimivirus genes were found to encode proteins unique amongst viruses and involved in DNA repair, protein folding, nucleotide synthesis, amino acid metabolism, protein modification, or lipid or polysaccharide metabolisms (Raoult et al., 2004). Moreover, messenger RNA were detected in Mimivirus capsids and the genome was found to encode transfer RNAs and to harbor late and early gene promoters (Raoult et al., 2004; Renesto et al., 2006; Legendre et al., 2010).

**Table 1:** **Major features of the main representatives of giant viruses of amoebas**.

**Family/Taxon**	**Sublineage**	**Prototype virus**		**Virion**		**Genome**		
		**Name**	**Genome GenBank Accession no**.	**Morphology**	**Size (nm)**	**Genome size (Kb)**	**Gene number**	**GC%**
*Mimiviridae*	A	*Acanthamoeba polyphaga* mimivirus	NC_014649.1	Icosahedral	≈750	1182	979	28.0
		Hirudovirus	KF493731.1	Icosahedral	≈630	1155	992	28.0
	B	*Acanthamoeba polyphaga* moumouvirus	NC_020104.1	Icosahedral	≈420	1021	930	24.6
	C	*Megavirus chiliensis*	NC_016072.1	Icosahedral	≈590	1259	1123	25.2
		LBA111 virus	NC_020232.1	Icosahedral	–	1231	1181	25.3
*Marseilleviridae*	A	*Marseillevirus marseillevirus*	NC_013756.1	Icosahedral	≈250	368	457	44.7
		Senegalvirus	JF909596.1-602.1	Icosahedral	≈250	373	479	–
	B	Lausannevirus	NC_015326.1	Icosahedral	≈250	347	444	42.9
	C	Tunisvirus	KF483846.1	Icosahedral	≈250	380	484	43.0
		Insectomime virus	KF527888.1	Icosahedral	≈250	387	477	42.7
*Pandoravirus*		*Pandoravirus salinus*	NC_022098.1	Ovoid	≈1000 × 500	2474	2544	61.7
		*Pandoravirus dulcis*	NC_021858.1	Ovoid	≈1000 × 500	1909	1488	63.7
		*Pandoravirus inopinatum*	NC_026440.1	Ovoid	≈1000 × 500	2243	1840	60.7
*Pithovirus*		*Pithovirus sibericum*	NC_023423.1	Ovoid	≈1500 × 500	610	467	35.8
*Faustovirus*	M	Faustovirus E12	KJ614390.1	Icosahedral	≈250	466	457	36.2
	D	Faustovirus D3	–	Icosahedral	≈250	456	495	37.8
	L	Faustovirus Liban	–	Icosahedral	≈250	471	518	36.7
	E9	Faustovirus E9	–	Icosahedral	≈250	491	511	39.6
*Mollivirus*		*Mollivirus sibericum*	NC_027867.1	Spheric	≈500–600	652	523	60.1
Virophages		Sputnik virophage	NC_011132.1	Icosahedral	≈50	18	21	27.0
		Zamilon virophage	NC_022990.1	Icosahedral	≈50	17	20	29.7

Dozens of mimiviruses were subsequently isolated using amoebal coculture, whose size, morphology, and genome are similar to those of Mimivirus (Arslan et al., 2011; Yoosuf et al., 2012; Pagnier et al., 2013; Figure 5). The capsid size of amoebal mimiviruses ranges between 370 and 600 nm in diameter. Amoebal mimivirus genomes are 1.02–1.26 Mbp in length, encode 930–1120 putative proteins, and are characterized by a high A+T content of ~72–75%. Phylogenomics divided these giant viruses in three lineages named A, led by Mimivirus; B, led by *A. polyphaga* moumouvirus (Yoosuf et al., 2012); and C, led by *Megavirus chiliensis* (Arslan et al., 2011; Table 1; Figures 1, 4, 5). In addition, distant mimivirus relatives were identified that infect marine organisms including phagocytic protists and microalgae, and encompass *Cafeteria roenbergensis* virus (Fischer et al., 2010), as well as Organic Lake phycodnaviruses (according to their primary classification; Yau et al., 2011), *Phaeocystis globosa* viruses (Santini et al., 2013), *Phaeocystis pouchetii* virus, *Chrysochromulina ericina* virus, and *Pyramimonas orientalis* virus (Yutin et al., 2013a,b).

High throughput strategies of isolation of giant viruses of amoebas carried out in Marseille led to the isolation to date of about 100 amoebal mimiviruses from various environmental water and soil samples, but also from human samples, which were collected in France, Tunisia, and Brazil, suggesting an ubiquitous distribution (Figure 5; Table 2) (Pagnier et al., 2013; Dornas et al., 2015). More recently, Hirudovirus, a mimivirus from the lineage A was isolated from a leech (Boughalmi et al., 2013a). Moreover, Lentillevirus was found by culturing the contact lens cleaning solution of a patient with keratitis, from which *A. polyphaga* co-infected by two other bacteria was also isolated (Cohen et al., 2011). Lastly, mimiviruses of lineage C were isolated from humans with pneumonia (Saadi et al., 2013a,b).

**Table 2:** **Major features of the main strains of mimiviruses**.

**Group**	**Lineage**	**Strain**	**Sample type**	**Sampling location**	**Additional information**	**References**
I	A	*Acanthamoeba polyphaga* mimivirus	Water of cooling tower	England		La Scola et al., 2003
I	A	*Acanthamoeba polyphaga* mamavirus	Water of cooling tower	France	Infected by the Sputnik virophage	Colson et al., 2011
I	A	Hirudovirus	Leech	France		Boughalmi et al., 2013a
I	A	Lentille virus	Contact lens cleaning solution	France	*Mycobacterium chelonae* and *Acanthamoeba polyphaga* co-infected by two other bacteria and a virophage were also isolated	Cohen et al., 2011
I	A	Samba virus	Negro River	Brazil	Infected by Rio Negro virophage	Campos et al., 2014
I	A	Niemeyer virus	Water sample of an urban lake	Brazil	–	Boratto et al., 2015
I	B	*Acanthamoeba polyphaga* moumouvirus	Cooling tower water	France	–	Yoosuf et al., 2012
I	C	*Megavirus chiliensis*	Ocean water off the coast	Chile	–	Arslan et al., 2011
I	C	LBA111	Broncho alveolar liquid	Tunisia	Patient with pneumonia	Saadi et al., 2013a
I	C	Courdo 11	Freshwater from a river	France	–	Yoosuf et al., 2014
I	C	Shan virus	Human stools	Tunisia	Patient with pneumonia	Saadi et al., 2013b
II	–	*Cafeteria roenbergensis* virus	Coastal waters	Texas	Infects a marine heterotrophic flagellate	Fischer et al., 2010

APMV was first identified during a pneumonia outbreak investigation while searching for resistant amoebal bacteria (La Scola et al., 2003; Raoult et al., 2007). The association between Mimivirus and pneumonia was strengthened by serological evidence. Thus, it was significant that patients with community- and hospital-acquired pneumonia exhibited antibodies to APMV more frequently than healthy controls, and seroconversions were observed (La Scola et al., 2005; Berger et al., 2006; Vincent et al., 2009; Bousbia et al., 2013; Colson et al., 2013c). Moreover, a Mimivirus seroconversion occurred in a 38-year-old technician who manipulated high amounts of Mimivirus (Raoult et al., 2006). He reported transfixing pain in his chest and bilateral basilar infiltrates suggesting viral pneumonia. Infections with the usual pneumonia agents were excluded, whereas seroconversion to 23 Mimivirus proteins was documented. Finally, in 2013, the first mimivirus isolated from a human was retrieved from the bronchoalveolar fluid of a Tunisian patient with unexplained pneumonia, and named LBA111 virus (Saadi et al., 2013a). Then, Shan virus, another lineage C mimivirus, was isolated from the feces of another patient with pneumonia (Saadi et al., 2013b). Moreover, in an experimental mouse model of infection, histopathological features of pneumonia, with thickened alveolar walls, inflammatory infiltrates, and diffuse alveolar damages, occurred after intracardiac Mimivirus inoculation (Khan et al., 2007).

Unexpectedly, Mimivirus isolations also led to the discovery of a new type of viruses, named virophages by analogy to bacteriophages (Table 1; Figures 1, 3–5; La Scola et al., 2008). Virophages cannot replicate alone in *Acanthamoeba* spp., but they replicate in the presence of a mimiviral host, in their viral factories (La Scola et al., 2008; Desnues and Raoult, 2012). Virophages have small (50 nm in diameter) virions with an icosahedral capsid and a dsDNA genome of ≈18,000 bp that encodes 20–21 proteins. The first virophage, named Sputnik, was described as infecting another strain of APMV, Mamavirus (La Scola et al., 2008). Sputnik replication impaired the normal replicative cycle and the morphogenesis of Mamavirus, decreasing by 70% the amoebal lysis and generating Mimivirus particles with abnormal morphologies. Three other Sputnik isolates were subsequently found, including Sputnik 2, which was co-isolated from a contact lens liquid with Lentillevirus, a mimivirus from lineage A (Cohen et al., 2011), and the Rio Negro virophage, which was isolated with Samba virus, a mimivirus recovered from the Negro river in Brazil (Campos et al., 2014). A divergent virophage of amoebal viruses, named Zamilon, was isolated from a soil sample collected in Tunisia with Mont1 virus, a mimivirus from lineage C, (Gaia et al., 2014). This virophage was capable of infecting mimiviruses from the B and C lineages, but not from the A lineage. Interestingly, antibodies against the Sputnik virophage were detected in two febrile patients returning from Laos, and seroconversion was noted in one case (Parola et al., 2012). Virophage DNA integration into the genome of mimiviruses, as pro-virophages, was demonstrated (Desnues et al., 2012). In addition, transposable elements, which were named transpovirons, were discovered in mimiviruses (Desnues et al., 2012). They are ~7 kb-long DNA elements that encode 6–8 proteins, among which two are homologous to virophage genes. They replicate in the mimivirus factory and accumulate inside virions, as well as in virophage particles and amoebas. Integrated genomes or fragments of transpovirons were detected in mimivirus and Sputnik DNA. Virophages, pro-virophages, and transpovirons comprise a mobilome in mimiviruses (Desnues et al., 2012). Other virophages were also described from distant mimiviruses, or phycodnaviruses (Zablocki et al., 2014; Blanc et al., 2015). Notably, a virophage named Mavirus was isolated with *C. roenbergensis* virus, a mimivirus deeply rooted in the branch of the family *Mimiviridae* (Fischer and Suttle, 2011).

## Marseilleviruses

In 2009, Marseillevirus was isolated in Marseille by co-culturing on *A. polyphaga*, from water collected in Paris in a cooling tower (Table 1; Figures 1–5) (Boyer et al., 2009). It had a 250 nm-large capsid with an icosahedral shape. Marseillevirus became the founding member of a new family of giant viruses of amoebas named *Marseilleviridae* (Colson et al., 2013d). Then, Lausannevirus (Thomas et al., 2011), the second member of this new family, and Cannes 8 virus (Aherfi et al., 2013), Tunisvirus (Aherfi et al., 2014), and Melbournevirus (Doutre et al., 2014), three other close relatives to Marseillevirus were isolated from the environment, including water samples collected from the Seine river, a cooling tower in Cannes (France), a decorative fountain in Tunis (Tunisia), and a pond in Melbourne (Australia), respectively. In addition, Insectomime virus was isolated from a diptera collected in Tunisia, and was the closest relative to Tunisvirus (Boughalmi et al., 2013b). Moreover, Senegalvirus was the first giant virus of amoeba isolated from a human sample, human feces collected from a young healthy man in Senegal and for which a metagenomic study serendipitously led to the generation and detection of marseillevirus-related reads (Lagier et al., 2012; Colson et al., 2013b).

The description of the Marseillevirus genome led to highlight a considerable genomic mosaicism, which has been suspected to be linked to the sympatric lifestyle in amoebas, where giant viruses can multiply in contact with other bacteria or fungi, and lateral genomic sequence transfers can occur (Raoult and Boyer, 2010). Marseillevirus genomes are 350–380 kilobp (kbp) large, with a G+C content of ≈45% and harbor genes they might have shared with bacteria, archaea, viruses, and eukaryotes, including amoebas (Aherfi et al., 2014). A surprising finding was the presence of genes encoding histone-like proteins and histone doublets, proteins only found so far in eukaryotic genomes (Boyer et al., 2009; Thomas et al., 2011). Notably, a recent report has suggested an evolutionary scenario in which marseillevirus core histones, as well as DNA topoisomerase II, would derive from a stem-eukaryotic lineage, which predates the neofunctionalization of histone paralogs of eukaryotes (Erives, 2015). In addition, large families of paralogous genes were found in marseillevirus genomes (Boyer et al., 2009; Aherfi et al., 2014). One of these families of genes encode proteins containing bacterial-like membrane occupation and recognition nexus (MORN) repeat domains. Moreover, marseillevirus genomes contain a very high number of family ORFans, i.e., genes only found in the family *Marseillevirid*ae when searching into the NCBI GenBank non redundant protein sequence database. Phylogenetic reconstruction based on the core genes of the *Megavirales* showed that the family *Marseilleviridae* encompasses three distinct lineages to date (Aherfi et al., 2014). The first one is led by Marseillevirus and contains close relatives, namely Cannes 8 virus, Senegalvirus, and Melbournevirus (Figure 5). Lausannevirus is the only representative of the second lineage. Lastly, the third lineage is comprised of Tunisvirus and Insectomime viruses.

After the Senegalvirus discovery from human feces, a metagenomic study of the human blood virome conducted in blood donors revealed the unexpected presence of a substantial number of sequences, representing 2.5% of the whole set of reads, that were related to Marseillevirus and could enable the assembly of two ≈10-kb-large contigs very similar to the Marseillevirus genome (Popgeorgiev et al., 2013a). Inoculation of the blood on Jurkat cells (human lymphocytes) enabled the replication, albeit not the propagation, of this virus that was named Giant Blood Marseillevirus. Subsequently, serological testing performed on 20 additional blood donors showed a high level of anti-Marseillevirus IgG for three of them, including two with a positive PCR for Marseillevirus. Moreover, seroprevalence studies showed seroprevalence rates ranging between 1.7 and 13% in the general population (Mueller et al., 2013) and up to 23% in polytransfused patients (Popgeorgiev et al., 2013b). Then, in 2013, a high titer of antibodies against Marseillevirus was serendipitously observed in one patient, while attempting to implement serological testing. This led to the detection of Marseillevirus by fluorescence *in situ* hybridization and immunohistochemistry in the lymph node from this patient, who was a 11-month-old child with a lymphadenitis (Popgeorgiev et al., 2013c).

## Pandoraviruses

From 2013, 10 and 5 years after the discoveries of Mimivirus and Marseillevirus, respectively, new giant viruses of amoebas were described and considerably expanded the phenotypic and genotypic diversity of this viral group. The two giant viral strains first described in 2013 were named *Pandoravirus salinus* and *Pandoravirus dulcis* (Table 1; Figures 1–5; Philippe et al., 2013). They were isolated by co-culturing on *Acanthamoeba castellanii* samples recovered from sediment layers collected in a river on a coast of central Chile and from mud collected from a freshwater pond close to Melbourne, Australia, respectively. Viral particles have ovoid shapes, with three layered membranes, and are 1 μm in length and 0.5 μm in diameter, which increased the size of the largest viral representative. As for Mimivirus that was thought for several years to be a Gram-positive bacterium (Raoult et al., 2007), pandoraviruses were previously described as *Acanthamoeba* parasites (Scheid et al., 2008). The genome from a third pandoravirus, *Pandoravirus inopinatum*, which was the one observed in 2008 but firstly mistaken as a parasite, was described in 2015 (Antwerpen et al., 2015).

The pandoravirus replicative cycle lasts 10–15 h, starting with the virus internalization through phagocytic vacuoles (Philippe et al., 2013). Viral particle contents are emptied via an apical pore and due to fusion of the viral internal lipid membrane with the vacuole membrane, which precedes the eclipse phase. Whereas, the replication cycle of APMV is entirely cytoplasmic, the cycle of pandoraviruses involves the reorganization of the host nucleus. Replication of the viral DNA and virion assembly occur simultaneously. New viral particles are released approximately after 10 h post-infection. Consistently with their exceptionally large particle sizes, *P. salinus* and *P. dulcis* were found to harbor the first and third largest viral genomes described so far, whose length are 2.45 and 1.91 Mbp, respectively. The genome tips harbor tandem repeats. The G+C-content is more than twice that of Mimivirus (61–64%; Philippe et al., 2013; Antwerpen et al., 2015). Interestingly, *P. dulcis* and *P. salinus*, the two first representatives of this new family have very different gene content sizes with 1502 and 2556 predicted genes, respectively (Philippe et al., 2013). Four large genomic fragments are specific to *P. salinus* compared to *P. dulcis*. The genome size of *P. inopinatum* (2.24 Mbp) is intermediate between those from the two other pandoravirus strains, and shows a nucleotide identity of 85 and 89% with genomes from *P. salinus* and *P. dulcis*, respectively (Antwerpen et al., 2015).

The *P. salinus* genome has a coding density of 80% (Philippe et al., 2013). A total of 401 genes (16% of the gene content) were shown to have known homologs in the GenBank non redundant protein sequence database, with a mean similarity of 38%. Among these genes, 54% contain ankyrin, MORN or F-box signatures. Best matches for the remaining genes were from eukaryotes, in nearly half of the cases, then from bacteria and viruses in equal proportions. Only 17 and 92 genes had Mimivirus and amoebozoa as best hit, respectively. Thus, strikingly, a large majority of the huge gene content of *P. salinus* has no homolog in the sequence databases. The *P. salinus* gene content includes 14 of the 31 class I-III core genes initially defined for the NCLDV (Iyer et al., 2001). Nonetheless, it is devoid of several core genes involved in DNA replication. More unexpectedly, no gene encoding a capsid protein was identified in pandoravirus genomes (Philippe et al., 2013; Yutin and Koonin, 2013). Previously, only a handful of smaller viruses had been identified as lacking a capsid (Koonin and Dolja, 2014). Approximately 10% of the *P. salinus* genes with homologs in the GenBank non redundant protein sequence database contain spliceosomal introns, which were described as differing from group I or II self-splicing introns found in other giant amoebal viruses (Philippe et al., 2013). Recently, 30 miniature inverted-repeat transposable elements (MITEs) were detected in the *P. salinus* genome, which might have been mobilized by an amoebal host (Sun et al., 2015). Proteomic analyses identified 210 *P. salinus* proteins in the virion, 80% being encoded by ORFans when searching for homologs in the GenBank non redundant protein sequence database, and none being a component of a transcription apparatus (Philippe et al., 2013). This latter finding, together with the detection of spliceosomal introns, suggested the significant implication of the *Acanthamoeba* nucleus in the viral replication, which was further bolstered by observations performed during the replicative cycle. Overall, pandoraviruses display remarkable features, including genomes mostly comprised of ORFans. Pandoraviruses are the most frequently clustered in phylogeny reconstructions with *Emiliana huxleyi* virus, a coccolithovirus, and it was suspected that they are highly derived phycodnaviruses; this remains controversial to date (Yutin and Koonin, 2013). Lastly, *P. inopinatum* was found to share 562 reciprocal best BLASTp hits with *P. salinus*, 510 with *P. dulcis*, while *P. salinus* and *P. dulcis* share 668 reciprocal best BLASTp hits (Sharma et al., 2015b). Among these bona fide orthologs, 375 are shared by these three pandoraviruses.

## Pithovirus sibericum

*P. sibericum* was described in 2014 and was recovered by co-culturing on *A. castellanii* with a ≈30,000-year-old Siberian permafrost sample collected in 2000 (Table 1; Figures 1, 3–5; Legendre et al., 2014). Virions have similar morphology as pandoraviruses but are still larger (1.5 μm in length × 0.5 μm in diameter), being the largest virions known so far. They have a structured envelope with a thickness of 60 nm. A hexagonal grid resembling structure, absent in pandoraviruses, closes the apical pore. The pithovirus replicative cycle lasts 10–20 h and is similar overall to that of pandoraviruses, apart from the absence of significant modification of the nucleus morphology. Mature virions appear after 6–8 h and may be released by exocytosis. Contrasting dramatically with a slightly larger virion size compared to pandoraviruses, the *P. sibericum* ds DNA genome is ~3–4 times shorter (610 kbp). In addition, the G+C-content of this genome is 36%, at mid-distance between those of mimiviruses and marseilleviruses, and almost half that of pandoraviruses. Genome conformation is either linear with terminal repeats, or circular. It was predicted to encode 467 proteins, of which 159 were detected inside the virion by proteomics, and two thirds of these protein sets are encoded by ORFans (as assessed by searching in the GenBank non redundant protein sequence database). BLAST matches were evenly distributed amongst bacteria, eukaryotes, and viruses, and among viruses between various *Megavirales* families. The greatest number of best viral matches was with marseilleviruses (19), then mimiviruses (15), and iridoviruses (10). These findings are consistent with phylogenetic and phyletic analyses that indicate a close evolutionary relationship of *P. sibericum* with marseilleviruses and iridoviruses (Legendre et al., 2014; Sharma et al., 2015a). Unexpectedly, similar amounts of proteins were detected in the virion than for *P. sibericum* and *P. salinus*, albeit these viruses have gene contents that differ dramatically in size.

One fifth of the *P. sibericum* genome corresponds to regularly interspersed copies of non-coding tandem repeats of a conserved palindromic pattern that is 150 bp-long and has a G+C content of 23%, similar to that of intergenic regions (compared to 41% for predicted genes). This feature lowers the genome coding density to 68%. A single group I self-splicing intron was detected, which is located inside the gene encoding the DNA dependent RNA polymerase subunit 1. No tRNA and component of the translation apparatus are encoded by the genome. In contrast, a comprehensive set of proteins involved in transcription was detected, which is consistent with cytoplasmic only replication. Only a very low similarity level was detected in the *P. sibericum* gene content with major capsid proteins, which belong to iridoviruses, and this pithovirus gene product was predicted to harbor a jelly-roll fold. As for pandoraviruses, pithovirus-ressembling endosymbionts had been previously reported inside *Acanthamoeba* (Michel et al., 2003).

## Faustoviruses

Faustovirus strain E12 was the first giant virus isolated on another free-living amoeba than *Acanthamoeba* spp. (Table 1; Figures 1–5; Reteno et al., 2015). The recovery of this virus from a sewage sample collected in Marseille, France, resulted from the implementation of a high-throughput strategy to isolate new giant viruses from environmental samples that included the use of five other protists, in addition to those from the genus *Acanthamoeba*, including *Vermamoeba vermiformis*, which appeared as the most common free-living protist in human environments (Bradbury, 2014). Faustovirus E12 was the prototype isolate to be described, then seven additional faustovirus isolates were recovered and their genomes sequenced (Reteno et al., 2015). Faustovirus virions have an icosahedral capsid with a diameter of 200 nm, and are devoid of fibers. These viruses are internalized inside *V. vermiformis* through phagocytosis. As for other *Megavirales* representatives, virions have an internal lipid membrane surrounding the core that fuses with the vacuole membrane to release the dsDNA genome. This seeds a cytoplasmic viral factory close to the host cell nucleus, which loses its regular morphology and shrinks. Approximately 10 h after infection, some amoebas show DNA-filled virions whereas others contain viral factories with only DNA-free capsids. The Faustovirus replicative cycle lasts 18–20 h post infection.

The Faustovirus E12 genome is a 466,265 bp long circular dsDNA. Its G+C content is 36%. With 451 genes predicted, its coding density is 85%. More than two thirds of these genes have no homologs in the GenBank non redundant protein sequence database whereas 13% have homologs in other *Megavirales* representatives, majoritarily in asfarviruses, then in phycodnaviruses, mimiviruses, marseilleviruses, and ascoviruses. In addition, other best hits are mostly sequences from bacteria (9%) and eukaryotes (7%), and, rarely (≈2%), sequences from archaea and phages. No tRNA gene was detected. One fifth of the gene content is comprised by paralogs, among which the most abundant are MORN-repeat containing proteins, previously first described among viruses in Marseillevirus (Boyer et al., 2009). Among the most remarkable genes were those encoding a ribosomal protein acetyltransferase, a bacteriophage tail fiber protein, and two polyproteins shared with asfarviruses (Reteno et al., 2015). Comparative genomics and phylogeny showed that faustoviruses were the most closely related, although distantly, to asfarviruses. Nonetheless, both groups of viruses have a distant evolutionary relationship and homologs represent only 12% of the faustovirus gene repertoire. Moreover, the size of the faustovirus genomes is approximately three times that of the asfarvirus genomes, and codon usage differs. Importantly, the core genome of faustoviruses and asfarviruses taken together is reduced 10-fold compared with the core genome of faustoviruses, which indicates large differences between the core genomes and gene contents of these two groups of viruses. Notably, phylogeny reconstruction based on the family B DNA polymerase showed that faustoviruses and asfarviruses were clustered with the *Heterocapsa circularisquama* virus, which infects dinoflagellates of marine water and whose genome is not available, but is thought to be a 356 kbp-long dsDNA (Ogata et al., 2009). One third of the Faustovirus E12 predicted proteins were detected inside its virions by proteomics (Reteno et al., 2015). An unexpected feature of Faustovirus was the architecture of a 17,000 kbp-long region harboring the capsid encoding genomic fragments, which appeared to be scattered along this region and separated from each other by non-coding regions interrupted by six group I self-splicing introns.

## Mollivirus sibericum

*M. sibericum* was isolated from the same 30,000-year-old Siberian permafrost sample as *P. sibericum* (Table 1; Figures 1, 3–5; Legendre et al., 2015). The virion has an original spheric shape, a diameter of 500–600 nm and is covered with 2–4 layers of fibers. Like the other giant viruses, it enters into amoeba by phagocytosis and fusion occurs between viral internal lipid and vacuole membranes, leading to genome release in the amoebal cytoplasm. Interestingly, the viral genome appears to enter into the amoebal nucleus, which is deformed. Then, neo-virions appear at its periphery, and are released after 6 h post-infection. These virions can be seen in vacuoles, suggesting an exocytosis pathway. Synthesis of the envelope and inner content of virions appears to occur simultaneously, as for pandoraviruses and *P. sibericum*. Strikingly, unlike for all other giant viruses of amoebas, viral factory is not cytoplasmic but perinuclear and the replication cycle is not lytic for the amoebal host.

The dsDNA genome of *M. sibericum* is linear, 651,523 bp in length and harbors inverted terminal repeats whose length is ~10 kbp. The G+C content is 60%, similar to that of pandoraviruses. A total of 523 genes and 3 tRNAs were predicted; no conserved promoter signal was identified in intergenic regions. Spliceosomal introns were detected in 4% of the genes. Approximately two thirds of the genes are ORFans. Among proteins with homologs, 18 and 3% were most similar to proteins from viruses and prokaryotes, respectively and 14% were most similar to eukaryotic proteins including 4% from *Acanthamoeba*. Among viral best hits, a large majority (90%) corresponded to pandoravirus sequences; they included the B family DNA polymerase and RNA polymerase II subunits 1 and 2. Fifty proteins were most similar to *Acanthamoeba*, a majority having an undetermined function. Among proteins with a functional annotation, the main group encompassed proteins containing ankyrin repeats, then proteins involved in DNA processing and nucleotide biosynthesis. However, thymidylate synthase and ribonucleotide reductase encoding genes were not detected. A total of 230 proteins were identified in virions, including 60% from *M. sibericum* and 40% from *Acanthamoeba*. No viral protein involved in transcription was detected. In contrast, unexpectedly, among *Acanthamoeba* encoded proteins detected inside virions, there were 23 ribosomal proteins from both large and small subunits, a ribosomal RNA assembly protein and a ribosome anti-association factor. Nevertheless, no intact ribosome could be seen inside virion. In addition, host encoded histone homologs and HMG-like chromatin-associated proteins were detected.

## Giant amoebal viruses as TRUC

A major controversy is whether giant amoebal viruses comprise a fourth branch in the tree of life, aside *Bacteria, Archaea*, and *Eukarya* (Boyer et al., 2010; Sharma et al., 2014). No gene has been detected that is shared by all viruses, and only five major viral groups could be shown being monophyletic (Koonin et al., 2006). Notably, viruses were overlooked in the classification of the living as they are devoid of ribosomal genes, which from the 1970s onwards became the most frequently considered molecular marker to build a tree of life (Woese et al., 1990). Therefore, due to their limited gene content, their subsequent strictly parasitic lifestyle with a replicative cycle that largely relies on the host proteins, and their invisibility under a light microscope, viruses long remained considered as being at the edge of the living world (Doermann, 1992; Raoult and Forterre, 2008).

At the time of Mimivirus genome description, Mimivirus was shown to branch near the origin of the eukaryotic domain in a phylogeny reconstruction based on concatenated sequences of seven conserved proteins (Raoult et al., 2004). Later, this observation has been reiterated by independent phylogenies based on informative and universally conserved genes, and strengthened based on hierarchical clustering that used the informational genes from the clusters of orthologous groups of proteins database (COG) and their giant viral homologs (Boyer et al., 2010; Sharma et al., 2015a,b). This fourth branch was not considered as an additional domain, since they were defined by Woese based on ribosomal genes that are missing in the giant viruses (Raoult and Forterre, 2008). Therefore, this new branch of life was named a fourth TRUC (an acronym for Things Resisting Uncompleted Classification; Raoult, 2013). TRUC corresponds to a new classification of microbes that divides the microbial world in four branches, including *Bacteria, Archaea, Eukarya*, and giant viruses. This reclassification allows including giant viruses in the tree of life, from which they are excluded in a classification centered on ribosomal DNA. This is further warranted as they are *bona fide* microbes, i.e., visible under an optical microscope, and have genomes that are larger than those of small bacteria and harbor an enormous gene content including homologs to cellular genes. The existence of a fourth branch of life was considered as unreliable by E. Koonin and his team, whose interpretation of their phylogenomic analyses is that giant viral genes were transferred from their eukaryotic hosts (Yutin et al., 2014), and by others who argued for lateral gene transfer from giant viral hosts, or artifactual results from phylogeny reconstructions (Moreira and Lopez-Garcia, 2009; Williams et al., 2011). In contrast, the fourth branch hypothesis was supported by data from other teams. Particularly, the analyses of protein fold superfamilies and their distribution among viruses and cellular organisms indicated that *Megavirales* representatives are grouped together, and apart from other viruses, while they overlap with some parasitic bacteria (Nasir et al., 2012; Nasir and Caetano-Anolles, 2015). These analyses further highlighted their ancestrality as they showed that giant viruses coexisted with the ancestors of cells and compose a distinct supergroup along with *Archaea, Bacteria*, and *Eukarya*. In addition, some sequences of the RecA superfamily or of DNA-dependent RNA polymerase generated by metagenomics from marine water stood between *Archaea, Bacteria*, and *Eukarya* in phylogeny reconstructions, and were suspected being from giant viruses (Wu et al., 2011).

Overall, megaviruses that infect amoebas exhibit remarkable features that place them on the edge of the viral world (Raoult, 2014). These giant viruses have virion sizes that are 2–15 times larger than traditional viruses, such as human immunodeficiency virus and hepatitis C virus, and genomes that contains ≈50–250 times more genes, among which large proportions are unique amongst viruses, and at least half have unknown functions. Moreover, they enter amoebas through phagocytosis (Clement et al., 2006; Ghigo et al., 2008; Ghigo, 2010; Backovic and Rey, 2012; Philippe et al., 2013; Legendre et al., 2014, 2015; Reteno et al., 2015). Hence, no specific interaction with cell receptors is needed, unlike for traditional viruses, and the giant size of amoebal viruses might be linked with this entry mechanism due to phagocytosis by these amoebas, as is the case for any particles larger than 0.5 microns (Raoult et al., 2007). It has been further suspected that, more generally, amoebas could be a training field for microorganisms to render them capable of entering human macrophages, and Mimivirus was demonstrated to enter these cells via phagocytosis (Ghigo et al., 2008; Salah et al., 2009).

## Giant amoebal viruses are common and highly diverse entities

Recently, the number and diversity of giant viruses of amoebas have expanded considerably (Table 1; Figures 1, 5), and it is likely that their diversity is still largely untapped. Their isolation has been recently boosted by using high throughput protocols and new protists as culture support (Pagnier et al., 2013; Reteno et al., 2015). These megaviruses, isolated using co-culturing on various amoebas and described over the last 12 years, display a wide range of virion sizes and shapes, structures, genome lengths, G+C%, gene repertoires and replicative sites (Table 1; Figure 3). Nevertheless, they comprise a monophyletic clade based on a limited set of core and informational genes (Yutin et al., 2009; Yutin and Koonin, 2012; Colson et al., 2013a; Sharma et al., 2015a). They were obtained from various environmental samples, ecosystems, and geographical locations, which indicate that they are common in our biosphere, and they were detected or isolated from amoebozoa, invertebrates or mammals (Colson and Raoult, 2012; Boughalmi et al., 2013a,b; Colson et al., 2013c; Pagnier et al., 2013; Popgeorgiev et al., 2013a; Dornas et al., 2014; Legendre et al., 2015; Figure 5; https://www.google.com/maps/d/edit?mid=zA3X4ljlz-uM.kFSrbnCtoBLc). Metagenomic data strengthen these observations as sequences related to amoebal megaviruses and virophages have been detected in several studies from environmental, animal, and human samples (Ghedin and Claverie, 2005; Monier et al., 2008; Loh et al., 2009; Kristensen et al., 2010; Colson et al., 2013b; Law et al., 2013; Zhang et al., 2015). Moreover, novel approaches to find sequences related to megaviruses in metagenomic datasets, using reconstructed putative ancestral sequences of conserved genes, can discover megaviruses previously overlooked (Sharma et al., 2014). Sequences from new putative giant viruses were also detected in marine environmental metagenomes (Wu et al., 2011; Mozar and Claverie, 2014) and in plant genomes (Maumus et al., 2014). Regarding *P. sibericum* and *M. sibericum*, recovered from 30,000-year-old permafrost samples, they are likely not viruses from ancient times that were revived. Indeed, metagenome sequences whose best matches were *P. sibericum* were recently detected, which suggests that close relatives to these viruses will probably be isolated in the near future. Lastly, the detection of mimiviruses and marseilleviruses in humans and accumulated hints of their potential pathogenicity is an emerging field (Colson et al., 2013c). This warrants investigating the presence and impact of all giant amoebal viruses in humans.

## Author contributions

All authors listed, have made substantial, direct and intellectual contribution to the work, and approved it for publication.

### Conflict of interest statement

The authors declare that the research was conducted in the absence of any commercial or financial relationships that could be construed as a potential conflict of interest.

## References

[B1] Abergel C. Rudinger-Thirion J. Giege R. Claverie J. M. (2007). Virus-encoded aminoacyl-tRNA synthetases: structural and functional characterization of mimivirus TyrRS and MetRS. J. Virol. 81, 12406–12417. 10.1128/JVI.01107-07 17855524 PMC2169003

[B2] Aherfi S. Boughalmi M. Pagnier I. Fournous G. La Scola B. Raoult D.. (2014). Complete genome sequence of Tunisvirus, a new member of the proposed family Marseilleviridae. Arch. Virol. 159, 2349–2358. 10.1007/s00705-014-2023-5 24770845

[B3] Aherfi S. Pagnier I. Fournous G. Raoult D. La Scola B. Colson P. (2013). Complete genome sequence of Cannes 8 virus, a new member of the proposed family “Marseilleviridae.” Virus Genes 47, 550–555. 10.1007/s11262-013-0965-4 23912978

[B4] Antwerpen M. H. Georgi E. Zoeller L. Woelfel R. Stoecker K. Scheid P. (2015). Whole-genome sequencing of a pandoravirus isolated from keratitis-inducing acanthamoeba. Genome Announc. 3, e00136–e00115. 10.1128/genomeA.00136-15 25814595 PMC4384135

[B5] Arslan D. Legendre M. Seltzer V. Abergel C. Claverie J. M. (2011). Distant Mimivirus relative with a larger genome highlights the fundamental features of Megaviridae. Proc. Natl. Acad. Sci. U.S.A. 108, 17486–17491. 10.1073/pnas.1110889108 21987820 PMC3198346

[B6] Backovic M. Rey F. A. (2012). Virus entry: old viruses, new receptors. Curr. Opin. Virol. 2, 4–13. 10.1016/j.coviro.2011.12.005 22440960 PMC7102732

[B7] Beijerinck M. W. (1898). Uber ein Contagium vivum fluidum als Ursache der Fleckenkrankheit der Tabaksblätter. Saint Paul, MN: American Phytopathological Society.

[B8] Berger P. Papazian L. Drancourt M. La Scola B. Auffray J. P. Raoult D. (2006). Ameba-associated microorganisms and diagnosis of nosocomial pneumonia. Emerging Infect. Dis. 12, 248–255. 10.3201/eid1202.050434 16494750 PMC3373093

[B9] Blanc G. Gallot-Lavallee L. Maumus F. (2015). Provirophages in the Bigelowiella genome bear testimony to past encounters with giant viruses. Proc. Natl. Acad. Sci. U.S.A. 112, E5318–E5326. 10.1073/pnas.1506469112 26305943 PMC4586850

[B10] Boratto P. V. Arantes T. S. Silva L. C. Assis F. L. Kroon E. G. La Scola B.. (2015). Niemeyer virus: a new mimivirus group A isolate harboring a set of duplicated aminoacyl-tRNA synthetase genes. Front. Microbiol. 6:1256. 10.3389/fmicb.2015.01256 26635738 PMC4639698

[B11] Boughalmi M. Pagnier I. Aherfi S. Colson P. Raoult D. La Scola B. (2013a). First isolation of a giant virus from wild Hirudo medicinalis leech: mimiviridae isolation in Hirudo medicinalis. Viruses 5, 2920–2930. 10.3390/v5122920 24287596 PMC3967153

[B12] Boughalmi M. Pagnier I. Aherfi S. Colson P. Raoult D. La Scola B. (2013b). First isolation of a marseillevirus in the diptera syrphidae *Eristalis tenax*. Intervirology 56, 386–394. 10.1159/000354560 24157885

[B13] Bousbia S. Papazian L. Saux P. Forel J. M. Auffray J. P. Martin C.. (2013). Serologic prevalence of amoeba-associated microorganisms in intensive care unit pneumonia patients. PLoS ONE 8:e58111. 10.1371/journal.pone.0058111 23469263 PMC3585915

[B14] Boyer M. Madoui M. A. Gimenez G. La Scola B. Raoult D. (2010). Phylogenetic and phyletic studies of informational genes in genomes highlight existence of a 4 domain of life including giant viruses. PLoS ONE 5:e15530. 10.1371/journal.pone.0015530 21151962 PMC2996410

[B15] Boyer M. Yutin N. Pagnier I. Barrassi L. Fournous G. Espinosa L.. (2009). Giant Marseillevirus highlights the role of amoebae as a melting pot in emergence of chimeric microorganisms. Proc. Natl. Acad. Sci. U.S.A. 106, 21848–21853. 10.1073/pnas.0911354106 20007369 PMC2799887

[B16] Bradbury R. S. (2014). Free-living amoebae recovered from human stool samples in Strongyloides agar culture. J. Clin. Microbiol. 52, 699–700. 10.1128/JCM.02738-13 24478518 PMC3911349

[B17] Campos R. K. Boratto P. V. Assis F. L. Aguiar E. R. Silva L. C. Albarnaz J. D.. (2014). Samba virus: a novel mimivirus from a giant rain forest, the Brazilian Amazon. Virol. J. 11:95. 10.1186/1743-422X-11-95 24886672 PMC4113263

[B18] Clement C. Tiwari V. Scanlan P. M. Valyi-Nagy T. Yue B. Y. Shukla D. (2006). A novel role for phagocytosis-like uptake in herpes simplex virus entry. J. Cell Biol. 174, 1009–1021. 10.1083/jcb.200509155 17000878 PMC2064392

[B19] Cohen G. Hoffart L. La Scola B. Raoult D. Drancourt M. (2011). Ameba-associated Keratitis, France. Emerg. Infect. Dis. 17, 1306–1308. 10.3201/eid1707.100826 21762597 PMC3381398

[B20] Colson P. de Lamballerie X. Yutin N. Asgari S. Bigot Y. Bideshi D. K.. (2013a). “Megavirales”, a proposed new order for eukaryotic nucleocytoplasmic large DNA viruses. Arch. Virol. 158, 2517–2521. 10.1007/s00705-013-1768-6 23812617 PMC4066373

[B21] Colson P. Fancello L. Gimenez G. Armougom F. Desnues C. Fournous G.. (2013b). Evidence of the megavirome in humans. J. Clin. Virol. 57, 191–200. 10.1016/j.jcv.2013.03.018 23664726

[B22] Colson P. La Scola B. Raoult D. (2013c). Giant viruses of amoebae as potential human pathogens. Intervirology 56, 376–385. 10.1159/000354558 24157884

[B23] Colson P. Pagnier I. Yoosuf N. Fournous G. La Scola B. Raoult D. (2013d). “Marseilleviridae”, a new family of giant viruses infecting amoebae. Arch. Virol. 158, 915–920. 10.1007/s00705-012-1537-y 23188494

[B24] Colson P. Raoult D. (2012). Megavirales composing a fourth domain of life: Mimiviridae and Marseilleviridae, in Viruses: Essential Agents of Life, ed Witzany G. (Dordretch: Springer), 217–244.

[B25] Colson P. Yutin N. Shabalina S. A. Robert C. Fournous G. La Scola B.. (2011). Viruses with more than 1,000 genes: Mamavirus, a new *Acanthamoeba polyphaga* mimivirus strain, and reannotation of Mimivirus genes. Genome Biol. Evol. 3, 737–742. 10.1093/gbe/evr048 21705471 PMC3163472

[B26] Desnues C. La Scola B. Yutin N. Fournous G. Robert C. Azza S.. (2012). Provirophages and transpovirons as the diverse mobilome of giant viruses. Proc. Natl. Acad. Sci. U.S.A. 109, 18078–18083. 10.1073/pnas.1208835109 23071316 PMC3497776

[B27] Desnues C. Raoult D. (2012). Virophages question the existence of satellites. Nat. Rev. Microbiol. 10, 234. 10.1038/nrmicro2676-c3 22337169

[B28] Doermann A. H. (1992). The eclipse in the bacteriophage life cycle, in Phage and the Origins of Molecular Biology, eds Cairns J. Stent G. S. Watson J. D. (Cold Spring Harbor, NY: Cold Spring Harbor Laboratory Press), 79–87.

[B29] Dornas F. P. Khalil J. Y. Pagnier I. Raoult D. Abrahao J. La Scola B. (2015). Isolation of new Brazilian giant viruses from environmental samples using a panel of protozoa. Front. Microbiol. 6:1086. 10.3389/fmicb.2015.01086 26500630 PMC4594340

[B30] Dornas F. P. Rodrigues F. P. Boratto P. V. Silva L. C. Ferreira P. C. Bonjardim C. A.. (2014). Mimivirus circulation among wild and domestic mammals, Amazon Region, Brazil. Emerg. Infect. Dis. 20, 469–472. 10.3201/eid2003.131050 24564967 PMC3944867

[B31] Doutre G. Philippe N. Abergel C. Claverie J. M. (2014). Genome analysis of the first Marseilleviridae representative from Australia indicates that most of its genes contribute to virus fitness. J. Virol. 88, 14340–14349. 10.1128/JVI.02414-14 25275139 PMC4249118

[B32] Edgar R. C. (2004). MUSCLE: multiple sequence alignment with high accuracy and high throughput. Nucleic Acids Res. 32, 1792–1797. 10.1093/nar/gkh340 15034147 PMC390337

[B33] Erives A. J. (2015). Eukaryotic core histone diversification in light of the histone doublet and DNA topo II genes of *Marseilleviridae*. bioRxiv. 10.1101/022236 PMC5704553 29179736

[B34] Fischer M. G. Allen M. J. Wilson W. H. Suttle C. A. (2010). Giant virus with a remarkable complement of genes infects marine zooplankton. Proc. Natl. Acad. Sci. U.S.A. 107, 19508–19513. 10.1073/pnas.1007615107 20974979 PMC2984142

[B35] Fischer M. G. Suttle C. A. (2011). A virophage at the origin of large DNA transposons. Science 332, 231–234. 10.1126/science.1199412 21385722

[B36] Gaia M. Benamar S. Boughalmi M. Pagnier I. Croce O. Colson P.. (2014). Zamilon, a novel virophage with Mimiviridae host specificity. PLoS ONE 9:e94923. 10.1371/journal.pone.0094923 24747414 PMC3991649

[B37] Ghedin E. Claverie J. M. (2005). Mimivirus relatives in the Sargasso sea. Virol. J. 2:62. 10.1186/1743-422X-2-62 16105173 PMC1215527

[B38] Ghigo E. (2010). A dilemma for viruses and giant viruses: which endocytic pathway to use to enter cells? Intervirology 53, 274–283. 10.1159/000312912 20551679

[B39] Ghigo E. Kartenbeck J. Lien P. Pelkmans L. Capo C. Mege J. L.. (2008). Ameobal pathogen mimivirus infects macrophages through phagocytosis. PLoS Pathog. 4:e1000087. 10.1371/journal.ppat.1000087 18551172 PMC2398789

[B40] Iyer L. M. Aravind L. Koonin E. V. (2001). Common origin of four diverse families of large eukaryotic DNA viruses. J. Virol. 75, 11720–11734. 10.1128/JVI.75.23.11720-11734.2001 11689653 PMC114758

[B41] Khan M. La Scola B. Lepidi H. Raoult D. (2007). Pneumonia in mice inoculated experimentally with *Acanthamoeba polyphaga* mimivirus. Microb. Pathog. 42, 56–61. 10.1016/j.micpath.2006.08.004 17188457

[B42] Klose T. Herbst D. A. Zhu H. Max J. P. Kenttamaa H. I. Rossmann M. G. (2015). A mimivirus enzyme that participates in viral entry. Structure 23, 1058–1065. 10.1016/j.str.2015.03.023 25982526 PMC4456301

[B43] Klose T. Rossmann M. G. (2014). Structure of large dsDNA viruses. Biol. Chem. 395, 711–719. 10.1515/hsz-2014-0145 25003382 PMC4307781

[B44] Koonin E. V. Dolja V. V. (2014). Virus world as an evolutionary network of viruses and capsidless selfish elements. Microbiol. Mol. Biol. Rev. 78, 278–303. 10.1128/MMBR.00049-13 24847023 PMC4054253

[B45] Koonin E. V. Senkevich T. G. Dolja V. V. (2006). The ancient virus world and evolution of cells. Biol. Direct. 1, 29. 10.1186/1745-6150-1-29 16984643 PMC1594570

[B46] Kristensen D. M. Mushegian A. R. Dolja V. V. Koonin E. V. (2010). New dimensions of the virus world discovered through metagenomics. Trends Microbiol. 18, 11–19. 10.1016/j.tim.2009.11.003 19942437 PMC3293453

[B47] La Scola B. Audic S. Robert C. Jungang L. de Lamballerie X. Drancourt M.. (2003). A giant virus in amoebae. Science 299, 2033. 10.1126/science.1081867 12663918

[B48] La Scola B. Desnues C. Pagnier I. Robert C. Barrassi L. Fournous G.. (2008). The virophage as a unique parasite of the giant mimivirus. Nature 455, 100–104. 10.1038/nature07218 18690211

[B49] La Scola B. Marrie T. J. Auffray J. P. Raoult D. (2005). Mimivirus in pneumonia patients. Emerg. Infect. Dis. 11, 449–452. 10.3201/eid1103.040538 15757563 PMC3298252

[B50] Lagier J. C. Armougom F. Million M. Hugon P. Pagnier I. Robert C.. (2012). Microbial culturomics: paradigm shift in the human gut microbiome study. Clin. Microbiol. Infect. 18, 1185–1193. 10.1111/1469-0691.12023 23033984

[B51] Law J. Jovel J. Patterson J. Ford G. O'Keefe S. Wang W.. (2013). Identification of hepatotropic viruses from plasma using deep sequencing: a next generation diagnostic tool. PLoS ONE 8:e60595. 10.1371/journal.pone.0060595 23613733 PMC3629200

[B52] Legendre M. Audic S. Poirot O. Hingamp P. Seltzer V. Byrne D.. (2010). mRNA deep sequencing reveals 75 new genes and a complex transcriptional landscape in Mimivirus. Genome Res. 20, 664–674. 10.1101/gr.102582.109 20360389 PMC2860168

[B53] Legendre M. Bartoli J. Shmakova L. Jeudy S. Labadie K. Adrait A.. (2014). Thirty-thousand-year-old distant relative of giant icosahedral DNA viruses with a pandoravirus morphology. Proc. Natl. Acad. Sci. U.S.A. 111, 4274–4279. 10.1073/pnas.1320670111 24591590 PMC3964051

[B54] Legendre M. Lartigue A. Bertaux L. Jeudy S. Bartoli J. Lescot M.. (2015). In-depth study of *Mollivirus sibericum*, a new 30,000-y-old giant virus infecting Acanthamoeba. Proc. Natl. Acad. Sci. U.S.A. 112, E5327–E5335. 10.1073/pnas.1510795112 26351664 PMC4586845

[B55] Loeffler F. Frosch P. (1898). Bericht der Kommission zur Erforschung der Maul- und Klauenseuche bei dem Institut für Infektionskrankheiten in Berlin. Centralblatt für Bakteriologie und Infektionskrankheiten. 2 naturwissenschaftenliche Abteilung: Allgemeine, land-wirtschaftliche-technologische Bakteriologie. Gärungsphysiol. Pflanzenphysiol. 23, 371–391.

[B56] Loh J. Zhao G. Presti R. M. Holtz L. R. Finkbeiner S. R. Droit L.. (2009). Detection of novel sequences related to african Swine Fever virus in human serum and sewage. J. Virol. 83, 13019–13025. 10.1128/JVI.00638-09 19812170 PMC2786824

[B57] Lwoff A. (1957). The concept of virus. J. Gen. Microbiol. 17, 239–253. 10.1099/00221287-17-2-239 13481308

[B58] Lwoff A. Tournier P. (1966). The classification of viruses. Annu. Rev. Microbiol. 20, 45–74. 10.1146/annurev.mi.20.100166.000401 5330240

[B59] Maumus F. Epert A. Nogue F. Blanc G. (2014). Plant genomes enclose footprints of past infections by giant virus relatives. Nat. Commun. 5, 4268. 10.1038/ncomms5268 24969138 PMC4083422

[B60] Michel R. Schmid E. N. Hoffmann R. Muller K. D. (2003). Endoparasite KC5/2 encloses large areas of sol-like cytoplasm within Acanthamoebae. Normal behavior or aberration? Parasitol. Res. 91, 265–266. 10.1007/s00436-003-0944-0 14574554

[B61] Monier A. Larsen J. B. Sandaa R. A. Bratbak G. Claverie J. M. Ogata H. (2008). Marine mimivirus relatives are probably large algal viruses. Virol. J. 5:12. 10.1186/1743-422X-5-12 18215256 PMC2245910

[B62] Moreira D. Lopez-Garcia P. (2009). Ten reasons to exclude viruses from the tree of life. Nat. Rev. Microbiol. 7, 306–311. 10.1038/nrmicro2108 19270719

[B63] Mozar M. Claverie J. M. (2014). Expanding the Mimiviridae family using asparagine synthase as a sequence bait. Virology 466–467, 112–122. 10.1016/j.virol.2014.05.013 24908633

[B64] Mueller L. Baud D. Bertelli C. Greub G. (2013). Lausannevirus seroprevalence among asymptomatic young adults. Intervirology 56, 430–433. 10.1159/000354565 24157889

[B65] Nasir A. Caetano-Anolles G. (2015). A phylogenomic data-driven exploration of viral origins and evolution. Sci. Adv. 1:e1500527. 10.1126/sciadv.1500527 26601271 PMC4643759

[B66] Nasir A. Kim K. M. Caetano-Anolles G. (2012). Giant viruses coexisted with the cellular ancestors and represent a distinct supergroup along with superkingdoms Archaea, Bacteria and Eukarya. BMC Evol. Biol. 12:156. 10.1186/1471-2148-12-156 22920653 PMC3570343

[B67] Ogata H. Toyoda K. Tomaru Y. Nakayama N. Shirai Y. Claverie J. M.. (2009). Remarkable sequence similarity between the dinoflagellate-infecting marine girus and the terrestrial pathogen African swine fever virus. Virol. J. 6:178. 10.1186/1743-422X-6-178 19860921 PMC2777158

[B68] Pagnier I. Reteno D. G. Saadi H. Boughalmi M. Gaia M. Slimani M.. (2013). A decade of improvements in Mimiviridae and Marseilleviridae isolation from amoeba. Intervirology 56, 354–363. 10.1159/000354556 24157882

[B69] Parola P. Renvoise A. Botelho-Nevers E. La Scola B. Desnues C. Raoult D. (2012). *Acanthamoeba polyphaga* mimivirus virophage seroconversion in travelers returning from Laos. Emerg. Infect. Dis. 18, 1500–1502. 10.3201/eid1809.120099 22932431 PMC3437713

[B70] Philippe N. Legendre M. Doutre G. Coute Y. Poirot O. Lescot M.. (2013). Pandoraviruses: amoeba viruses with genomes up to 2.5 Mb reaching that of parasitic eukaryotes. Science 341, 281–286. 10.1126/science.1239181 23869018

[B71] Popgeorgiev N. Boyer M. Fancello L. Monteil S. Robert C. Rivet R.. (2013a). Giant Blood Marseillevirus recovered from asymptomatic blood donors. J. Infect. Dis. 208, 1042–1050. 10.1093/infdis/jit292 23821720

[B72] Popgeorgiev N. Colson P. Thuret I. Chiarioni J. Gallian P. Raoult D.. (2013b). Marseillevirus prevalence in multitransfused patients suggests blood transmission. J. Clin. Virol. 58, 722–725. 10.1016/j.jcv.2013.10.001 24183312

[B73] Popgeorgiev N. Michel G. Lepidi H. Raoult D. Desnues C. (2013c). Marseillevirus adenitis in an 11-month-old child. J. Clin. Microbiol. 51, 4102–4105. 10.1128/JCM.01918-13 24088856 PMC3838047

[B74] Price M. N. Dehal P. S. Arkin A. P. (2010). FastTree 2–approximately maximum-likelihood trees for large alignments. PLoS ONE 5:e9490. 10.1371/journal.pone.0009490 20224823 PMC2835736

[B75] Raoult D. (2013). TRUC or the need for a new microbial classification. Intervirology 56, 349–353. 10.1159/000354269 23867259

[B76] Raoult D. (2014). How the virophage compels the need to readdress the classification of microbes. Virology 477, 119–124. 10.1016/j.virol.2014.11.014 25497204

[B77] Raoult D. Audic S. Robert C. Abergel C. Renesto P. Ogata H.. (2004). The 1.2-megabase genome sequence of Mimivirus. Science 306, 1344–1350. 10.1126/science.1101485 15486256

[B78] Raoult D. Boyer M. (2010). Amoebae as genitors and reservoirs of giant viruses. Intervirology 53, 321–329. 10.1159/000312917 20551684

[B79] Raoult D. Forterre P. (2008). Redefining viruses: lessons from Mimivirus. Nat. Rev. Microbiol. 6, 315–319. 10.1038/nrmicro1858 18311164

[B80] Raoult D. La Scola B. Birtles R. (2007). The discovery and characterization of Mimivirus, the largest known virus and putative pneumonia agent. Clin. Infect. Dis. 45, 95–102. 10.1086/518608 17554709

[B81] Raoult D. Renesto P. Brouqui P. (2006). Laboratory infection of a technician by mimivirus. Ann. Intern. Med. 144, 702–703. 10.7326/0003-4819-144-9-200605020-00025 16670147

[B82] Renesto P. Abergel C. Decloquement P. Moinier D. Azza S. Ogata H.. (2006). Mimivirus giant particles incorporate a large fraction of anonymous and unique gene products. J. Virol. 80, 11678–11685. 10.1128/JVI.00940-06 16971431 PMC1642625

[B83] Reteno D. G. Benamar S. Boukhalil J. Andreani J. Armstrong N. Klose T.. (2015). Faustovirus, an asfarvirus-related new lineage of giant viruses infecting amoebae. J. Virol. 89, 6585–6589. 10.1128/JVI.00115-15 25878099 PMC4468488

[B84] Rodrigues R. A. Silva L. K. Dornas F. P. de Oliveira D. B. Magalhaes T. F. Santos D. A.. (2015). Mimivirus fibrils are important for viral attachment to microbial world by a diverse glycoside interaction repertoire. J. Virol. 89, 11812–11819. 10.1128/JVI.01976-15 26378162 PMC4645322

[B85] Rowbotham T. J. (1983). Isolation of Legionella pneumophila from clinical specimens via amoebae, and the interaction of those and other isolates with amoebae. J. Clin. Pathol. 36, 978–986. 10.1136/jcp.36.9.978 6350372 PMC498455

[B86] Saadi H. Pagnier I. Colson P. Cherif J. K. Beji M. Boughalmi M.. (2013a). First isolation of Mimivirus in a patient with pneumonia. Clin. Infect. Dis. 57, e127–e134. 10.1093/cid/cit354 23709652

[B87] Saadi H. Reteno D. G. Colson P. Aherfi S. Minodier P. Pagnier I.. (2013b). Shan virus: a new mimivirus isolated from the stool of a Tunisian patient with pneumonia. Intervirology 56, 424–429. 10.1159/000354564 24157888

[B88] Salah I. B. Ghigo E. Drancourt M. (2009). Free-living amoebae, a training field for macrophage resistance of mycobacteria. Clin. Microbiol. Infect. 15, 894–905. 10.1111/j.1469-0691.2009.03011.x 19845701

[B89] Santini S. Jeudy S. Bartoli J. Poirot O. Lescot M. Abergel C.. (2013). Genome of *Phaeocystis globosa* virus PgV-16T highlights the common ancestry of the largest known DNA viruses infecting eukaryotes. Proc. Natl. Acad. Sci. U.S.A. 110, 10800–10805. 10.1073/pnas.1303251110 23754393 PMC3696832

[B90] Scheid P. Zoller L. Pressmar S. Richard G. Michel R. (2008). An extraordinary endocytobiont in Acanthamoeba sp. isolated from a patient with keratitis. Parasitol. Res. 102, 945–950. 10.1007/s00436-007-0858-3 18210154

[B91] Sharma V. Colson P. Chabrol O. Pontarotti P. Raoult D. (2015a). *Pithovirus sibericum*, a new bona fide member of the “Fourth TRUC” club. Front. Microbiol. 6:722. 10.3389/fmicb.2015.00722 26300849 PMC4523831

[B92] Sharma V. Colson P. Chabrol O. Scheid P. Pontarotti P. Raoult D. (2015b). Welcome to pandoraviruses at the ‘Fourth TRUC’ club. Front. Microbiol. 6:423. 10.3389/fmicb.2015.00423 26042093 PMC4435241

[B93] Sharma V. Colson P. Giorgi R. Pontarotti P. Raoult D. (2014). DNA-dependent RNA polymerase detects hidden giant viruses in published databanks. Genome Biol. Evol. 6, 1603–1610. 10.1093/gbe/evu128 24929085 PMC4122926

[B94] Silva L. C. Almeida G. M. Assis F. L. Albarnaz J. D. Boratto P. V. Dornas F. P.. (2015). Modulation of the expression of mimivirus-encoded translation-related genes in response to nutrient availability during *Acanthamoeba castellanii* infection. Front. Microbiol. 6:539. 10.3389/fmicb.2015.00539 26082761 PMC4450173

[B95] Sun C. Feschotte C. Wu Z. Mueller R. L. (2015). DNA transposons have colonized the genome of the giant virus *Pandoravirus salinus*. BMC Biol. 13:38. 10.1186/s12915-015-0145-1 26067596 PMC4495683

[B96] Thomas V. Bertelli C. Collyn F. Casson N. Telenti A. Goesmann A.. (2011). Lausannevirus, a giant amoebal virus encoding histone doublets. Environ. Microbiol. 13, 1454–1466. 10.1111/j.1462-2920.2011.02446.x 21392201

[B97] Vincent A. La Scola B. Forel J. M. Pauly V. Raoult D. Papazian L. (2009). Clinical significance of a positive serology for mimivirus in patients presenting a suspicion of ventilator-associated pneumonia. Crit. Care Med. 37, 111–118. 10.1097/CCM.0b013e318192fa8b 19050618

[B98] Williams T. A. Embley T. M. Heinz E. (2011). Informational gene phylogenies do not support a fourth domain of life for nucleocytoplasmic large DNA viruses. PLoS ONE 6:e21080. 10.1371/journal.pone.0021080 21698163 PMC3116878

[B99] Woese C. R. Kandler O. Wheelis M. L. (1990). Towards a natural system of organisms: proposal for the domains Archaea, Bacteria, and Eucarya. Proc. Natl. Acad. Sci. U.S.A. 87, 4576–4579. 10.1073/pnas.87.12.4576 2112744 PMC54159

[B100] Wu D. Wu M. Halpern A. Rusch D. B. Yooseph S. Frazier M.. (2011). Stalking the fourth domain in metagenomic data: searching for, discovering, and interpreting novel, deep branches in marker gene phylogenetic trees. PLoS ONE 6:e18011. 10.1371/journal.pone.0018011 21437252 PMC3060911

[B101] Yau S. Lauro F. M. DeMaere M. Z. Brown M. V. Thomas T. Raftery M. J.. (2011). Virophage control of antarctic algal host-virus dynamics. Proc. Natl. Acad. Sci. U.S.A. 108, 6163–6168. 10.1073/pnas.1018221108 21444812 PMC3076838

[B102] Yoosuf N. Yutin N. Colson P. Shabalina S. A. Pagnier I. Robert C.. (2012). Related giant viruses in distant locations and different habitats: *Acanthamoeba polyphaga* moumouvirus represents a third lineage of the Mimiviridae that is close to the megavirus lineage. Genome Biol. Evol. 4, 1324–1330. 10.1093/gbe/evs109 23221609 PMC3542560

[B103] Yoosuf N. Pagnier I. Fournous G. Robert C. La Scola B. Raoult D.. (2014). Complete genome sequence of Courdo11 virus, a member of the family *Mimiviridae*. Virus Genes 48, 218–223. 10.1007/s11262-013-1016-x 24293219

[B104] Yutin N. Colson P. Raoult D. Koonin E. V. (2013a). Mimiviridae: clusters of orthologous genes, reconstruction of gene repertoire evolution and proposed expansion of the giant virus family. Virol. J. 10, 106. 10.1186/1743-422X-10-106 23557328 PMC3620924

[B105] Yutin N. Koonin E. V. (2012). Hidden evolutionary complexity of Nucleo-Cytoplasmic Large DNA viruses of eukaryotes. Virol. J. 9:161. 10.1186/1743-422X-9-161 22891861 PMC3493329

[B106] Yutin N. Koonin E. V. (2013). Pandoraviruses are highly derived phycodnaviruses. Biol. Direct. 8, 25–28. 10.1186/1745-6150-8-25 24148757 PMC3924356

[B107] Yutin N. Raoult D. Koonin E. V. (2013b). Virophages, polintons, and transpovirons: a complex evolutionary network of diverse selfish genetic elements with different reproduction strategies. Virol. J. 10:158. 10.1186/1743-422X-10-158 23701946 PMC3671162

[B108] Yutin N. Wolf Y. I. Koonin E. V. (2014). Origin of giant viruses from smaller DNA viruses not from a fourth domain of cellular life. Virology 466–467, 38–52. 10.1016/j.virol.2014.06.032 25042053 PMC4325995

[B109] Yutin N. Wolf Y. I. Raoult D. Koonin E. V. (2009). Eukaryotic large nucleo-cytoplasmic DNA viruses: clusters of orthologous genes and reconstruction of viral genome evolution. Virol. J. 17:223. 10.1186/1743-422X-6-223 20017929 PMC2806869

[B110] Zablocki O. van Zyl L. Adriaenssens E. M. Rubagotti E. Tuffin M. Cary S. C.. (2014). High-level diversity of tailed phages, eukaryote-associated viruses, and virophage-like elements in the metaviromes of antarctic soils. Appl. Environ. Microbiol. 80, 6888–6897. 10.1128/AEM.01525-14 25172856 PMC4249006

[B111] Zhang W. Zhou J. Liu T. Yu Y. Pan Y. Yan S.. (2015). Four novel algal virus genomes discovered from Yellowstone Lake metagenomes. Sci. Rep. 5:15131. 10.1038/srep15131 26459929 PMC4602308

